# Removal of silver nanoparticles by mussel-inspired Fe_3_O_4_@ polydopamine core-shell microspheres and its use as efficient catalyst for methylene blue reduction

**DOI:** 10.1038/srep42773

**Published:** 2017-02-16

**Authors:** Maoling Wu, Yinying Li, Rui Yue, Xiaodan Zhang, Yuming Huang

**Affiliations:** 1 The Key Laboratory of Eco-environments in Three Gorges Reservoir Region, Ministry of Education, College of Chemistry and Chemical Engineering, Southwest University, Chongqing 400715, China

## Abstract

The removal of silver nanoparticles (AgNPs) from water is highly needed because of their increasing use and potential risk to the environment due to their toxic effects. Catalysis over AgNPs has received significant attention because of their highly catalytic performance. However, their use in practical applications is limited due to high cost and limited resources. Here, we present for the first time that the mussel-inspired Fe_3_O_4_@polydopamine (Fe_3_O_4_@PDA) nanocomposite can be used for efficient removal and recovery of AgNPs. Adsorption of AgNPs over Fe_3_O_4_@PDA was confirmed by TEM, FT-IR, XRD, TGA and magnetic property. The adsorption efficiency of AgNPs by Fe_3_O_4_@PDA was investigated as a function of pH, contact time, ionic strength and concentration of AgNPs. The kinetic data were well fitted to a pseudo-second order kinetic model. The isotherm data were well described by Langmuir model with a maximum adsorption capacity of 169.5 mg/g, which was higher than those by other adsorbents. Notably, the obtained AgNPs-Fe_3_O_4_@PDA exhibited highly catalytic activity for methylene blue reduction by NaBH_4_ with a rate constant of 1.44 × 10^−3^/s, which was much higher than those by other AgNPs catalysts. The AgNPs-Fe_3_O_4_@PDA promised good recyclability for at least 8 cycles and acid resistant with good stability.

The engineered nanoparticles (NPs) are increasingly used in industrial and consumer products due to their unique properties. Among engineered NPs, silver NPs (AgNPs) are one of the commercially used nanomaterials in detergents, plastics, and textiles due to their excellent antimicrobial properties^1^,^2^,^3^. The production and the increasing use of AgNPs obviously results in their release into the environment, leading to a risk to the environment due to their toxic effects^4^,^5^. Thus, the removal of AgNPs from water is urgently needed.

To date, a variety of approaches such as filtration^6^, cloud point extraction^7^,^8^, and adsorption technology^9^,^10^,^11^,^12^,^13^,^14^,^15^,^16^,^17^,^18^, have been developed for the separation and extraction of metal NPs from water. Among these, adsorption is a promising one for the removal of AgNPs from aqueous solution due to its low cost and ease operation. For example, Valiyaveettil’s group used biomimetic metal oxides^10^, cellulose and polyvinyl alcohol/gluten-based nanofibers^11^,^12^,^13^, polyethyleneimine modified carbon spheres^14^ and amine-modified block copolymers^15^ for adsorption of AgNPs from water. Khan *et al*. illustrated the potential of the resistant bacterial species *Aeromonas punctata* for the effective removal of AgNPs^16^. More recently, the group of Černík reported the use of methane plasma treated electrospun nanofibres for the removal of various NPs in water^17^,^18^. Above studies demonstrate the high potential of adsorption technology for the effective removal of the engineered NPs.

On the other hand, the noble metallic NPs have been received great attention in the field of catalysis due to their superior chemical and physical properties. In particular, catalysis over AgNPs has received the most attention due to their highly catalytic performance^19^,^20^,^21^,^22^. However, their use in practical applications is highly limited due to high cost and limited resources. Therefore, from the view point of economic and environmental reasons, recovery and reuse of expensive noble metal catalysts play a key role in both heterogeneous and homogenous catalysis. However, no attempts have been made to explore the magnetic separation of AgNPs from aqueous media as a new strategy toward stable and recyclable catalyst for the reduction of toxic pollutants.

Here, for the first time, we reported a novel magnetic Fe_3_O_4_@polydopamine (Fe_3_O_4_@PDA) core-shell microsphere for the removal of AgNPs from the aqueous solution, and the obtained AgNPs-Fe_3_O_4_@PDA was used as catalyst for the reduction of methylene blue (MB) by NaBH_4_. As compared to other technologies, the magnetic separation removal of pollutants from water promises advantages of low-cost Fe_3_O_4_ as raw materials, easy to prepare and scale up, and easy separation from aqueous solution by a magnetic field^23^. PDA is utilized because it is a versatile and intriguing starting material for solid surface modification and autopolymerized to form PDA under mild conditions^24^,^25^, and has advantages such as: (1) a robust interfacial binding force between the coating and the substrate through covalent bonds and other strong intermolecular interactions^26^; (2) the PDA coatings are stable even in harsh environments such as a strong acid or alkaline solution; (3) PDA coatings have good hydrophilic and biocompatible properties. Our results reveal that Fe_3_O_4_@PDA composite is a promising adsorbent for the extraction and recovery of AgNPs with a maximum adsorptive capacity of 169.5 mg/g. Interestingly, the obtained AgNPs-Fe_3_O_4_@PDA was shown to exhibit highly catalytic ability for MB reduction by NaBH_4_. More importantly, the AgNPs-Fe_3_O_4_@PDA holds excellent cyclic performance via magnetic separation and can be reused for more than eight times, showing good potentials in practical applications. Further, the AgNPs-Fe_3_O_4_@PDA is acid resistant, showing good stability.

## Results and Discussion

### Synthesis and characterization of Fe_3_O_4_@PDA

**Figure 1:**
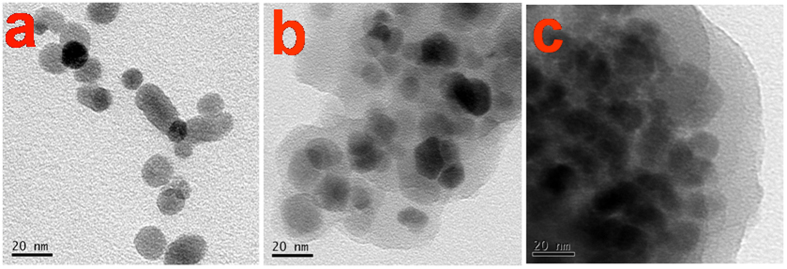
TEM images of (**a**) Fe_3_O_4_ NPs, (**b**) Fe_3_O_4_@PDA core-shell NPs and (**c**) AgNPs-Fe_3_O_4_@PDA.

**Figure 2:**
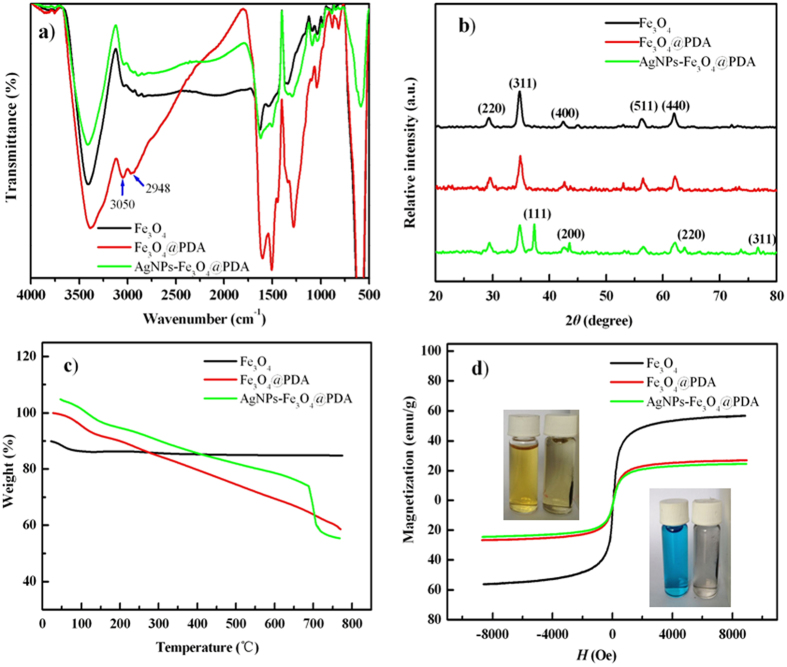
(**a**) FT-IR spectra of Fe_3_O_4_, Fe_3_O_4_@PDA, and AgNPs-Fe_3_O_4_@PDA. (**b**) X-ray diffraction patterns of Fe_3_O_4_, Fe_3_O_4_@PDA, and AgNPs-Fe_3_O_4_@PDA. (**c**) TGA data of Fe_3_O_4_, Fe_3_O_4_@PDA, and AgNPs-Fe_3_O_4_@PDA. (**d**) Magnetic curves of Fe_3_O_4_, Fe_3_O_4_@PDA, and AgNPs-Fe_3_O_4_@PDA, inset photographs: separation of Fe_3_O_4_@PDA (upper left) and AgNPs-Fe_3_O_4_@PDA (lower right) from aqueous solution by using an external magnet.

The Fe_3_O_4_@PDA was facilely prepared by direct coating of PDA onto the surface of Fe_3_O_4_ via a simple one-step reaction. The Fe_3_O_4_ NPs were nearly spherical with a diameter of 10~20 nm (Fig. 1a) and the PDA shell layers formed around the Fe_3_O_4_ cores (Fig. 1b). A clear aggregation was formed on the surface of Fe_3_O_4_@PDA after adding of AgNPs suspension, indicating successful adsorption of AgNPs by Fe_3_O_4_@PDA (Fig. 1c), which was confirmed by the results of FT-IR spectra and XRD. Fe_3_O_4_@PDA exhibited bands at 3050 cm^−1^ and 2948 cm^−1^ (Fig. 2a), attributed to N-H, C-H vibration peak of benzene. The additional bands at 1000–1700 cm^−1^ may be ascribed to the aromatic rings of PDA and the amide I, amide II and C-N stretching bands^27^,^28^. These peaks weakened or disappeared after adsorption of gum arabic (GA) capped AgNPs (GA-AgNPs), indicating that AgNPs interacts with O and N atoms of PDA during adsorption process. A similar XRD pattern to Fe_3_O_4_ was observed for the Fe_3_O_4_@PDA (Fig. 2b), indicating that the crystalline structure of Fe_3_O_4_ was not affected by coating with PDA. The diffraction peaks of AgNPs-Fe_3_O_4_@PDA can be indexed to Ag (JCPDS 04-0783), confirming the presence of AgNPs in the Fe_3_O_4_@PDA^29^,^30^. TG data (Fig. 2c) indicate the weight loss of water from 0 to 100 °C. Above 100 °C, Fe_3_O_4_ NPs were very stable, while Fe_3_O_4_@PDA and AgNPs-Fe_3_O_4_@PDA had about 15% loss of weight. This may be due to the part weight loss of PDA coating. At 700 °C, AgNPs-Fe_3_O_4_@PDA had another 15% loss of weight due to the part weight loss of GA. The result confirms the successful synthesis and excellent thermal stability of Fe_3_O_4_@PDA as well as adsorption of GA-AgNPs by Fe_3_O_4_@PDA. The saturation magnetization values were 60.4, 27.5, and 25.9 emu/g for Fe_3_O_4_, Fe_3_O_4_@PDA and AgNPs-Fe_3_O_4_@PDA, respectively (Fig. 2d). Compared with Fe_3_O_4_, the decrease in saturation magnetization value of the Fe_3_O_4_@PDA was mainly due to the coating of Fe_3_O_4_ with PDA. However, no obvious loss of saturation magnetization was observed after adsorption of GA-AgNPs by Fe_3_O_4_@PDA. Hence, the AgNPs-Fe_3_O_4_@PDA can be easily separated from solution by an external magnetic field (inset in Fig. 2d).

In this study, GA capped AgNPs was used as a target to investigate its magnetic separation by Fe_3_O_4_@PDA because GA-AgNPs was found in commercial products of AgNPs^31^. Supplementary Figure 1a and b show the TEM image and corresponding size distribution histogram of the synthesized GA-AgNPs, respectively. GA-AgNPs were sphere-like with an average diameter of 5.0 ± 1.7 nm based on the statistic result of 172 particles.

### Adsorption of the prepared adsorbents for AgNPs

After PDA coating, the dynamic uptake capacity of the Fe_3_O_4_@PDA for GA-AgNPs increased by 7-fold compared with that of Fe_3_O_4_ (Supplementary Figure 2). The result indicates that the Fe_3_O_4_ core plays the role of magnetic separation and the PDA shell contributs mainly to the adsorption removal of GA-AgNPs. To understand the possible adsorption mechanism, the zeta potentials of the synthesized GA-AgNPs and Fe_3_O_4_@PDA were measured. In pH 10 solution, GA-AgNP is negatively charged and its zeta potential is −25.0 mV. While Fe_3_O_4_@PDA is also negatively charged and its zeta potential is −43.6 mV at pH 10. This indicates that the electrostatic force is not the main driving force for the adsorption of GA-AgNPs by Fe_3_O_4_@PDA. In contrast, the specific high affinity of AgNPs to PDA through its complexation with catechol group^28^ on Fe_3_O_4_@PDA surface may be responsible for adsorption separation of GA-AgNPs. In addition, the Fe_3_O_4_@PDA is suitable for removal of AgNPs capped with other capping agents, including polyvinyl alcohol (PVA), polyvinylpyrrolidone (PVP), humic acid (HA), citrate (Cit) and polyethyleneimine (PEI) (Supplementary Table 1). It is noted that the adsorption capacity of Fe_3_O_4_@PDA for AgNPs depends on the used capping agents. This may be due to the difference in the zeta potential and hydrodynamic diameter of the obtained AgNPs. However, the effects of the zeta potential and hydrodynamic diameter of the obtained AgNPs on adsorption capacity of Fe_3_O_4_@PDA for AgNPs did not show a clear trend. Also, the Fe_3_O_4_@PDA works for removal of AuNPs capped with GA, PVP and Cit (Supplementary Figure 3).

### Effect of pH

**Figure 3:**
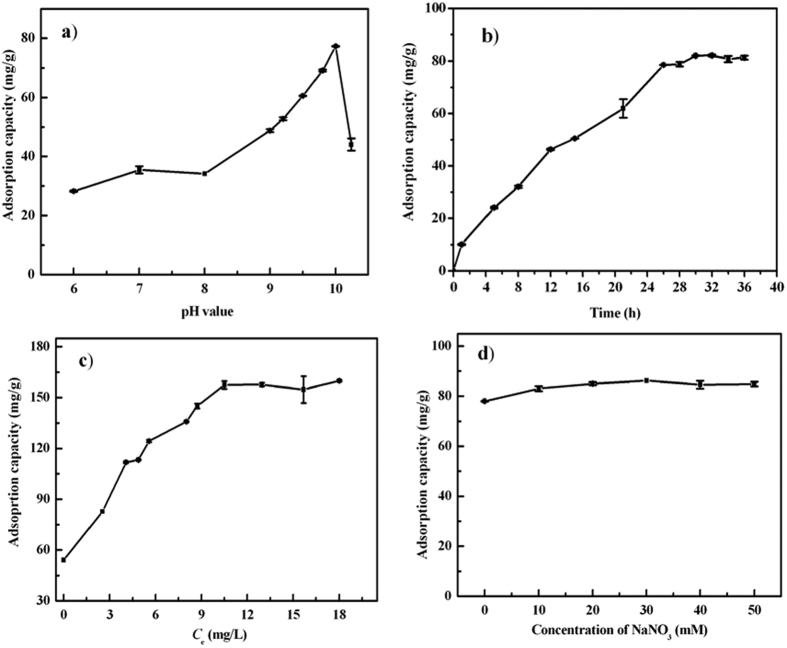
(**a**) Effect of pH. Reaction conditions: 5 mg adsorbent, 50 mL of 10.8 mg/L GA-AgNPs solution, adsorption time 26 h. (**b**) Adsorption kinetics. Reaction conditions: 5 mg adsorbent, 50 mL of 10.8 mg/L GA-AgNPs solution, pH 10.0. (**c**) Adsorption isotherm. Reaction conditions: 5 mg adsorbent, 50 mL of GA-AgNPs solution, pH 10.0, and adsorption time 26 h. (**d**) Effect of salt concentration. Reaction conditions: 5 mg adsorbent, 50 mL of 10.8 mg/L GA-AgNPs solution, pH 10.0, adsorption time 26 h. Error bars represent one standard deviation for three measurements.

The effect of pH on the adsorption of AgNPs to Fe_3_O_4_@PDA was explored. The adsorption of AgNPs increased with the increase of pH to 10, above which it decreased (Fig. 3a). This can be explained by the formation of Ag–catechol bonds through the specific high affinity between AgNPs with PDA on the surface of Fe_3_O_4_^32^. It has been reported that the amino and phenolic hydroxyl groups in the PDA coating of PDA@Fe_3_O_4_ particles are expected to be deprotonated in the pH range of 8.0–10.0^33^. At pH < 8, minor –OH groups are ionized to form –O^−^ groups, and the adsorption capacity of AgNPs keeps minor change in pH range from 6 to 8. While at pH > 8, more –OH groups of PDA are ionized to form –O^−^ groups with increase in solution pH due to deprotonation of amino and phenolic hydroxyl groups in the PDA coating, and the adsorption of AgNPs increased with increase in solution pH from 8 to 10. However, higher pH above 10 caused decrease in the adsorption of AgNPs. This is probably attributed to hinderation of approach of the negatively charged AgNPs to the PDA surface in high pH solution^33^.

### Adsorption kinetics and adsorption isotherms

The time-dependent adsorption capacity was obtained to study the kinetics of GA-AgNPs adsorption on Fe_3_O_4_@PDA. The sorption rate of GA-AgNPs onto Fe_3_O_4_@PDA increased with increase in contact time to 26 h, and then the adsorption capacity increased slightly with contact time up to 36 h (Fig. 3b). Thus, the sorption process for Fe_3_O_4_@PDA reaches equilibrium at about 26 h. A better fit of the pseudo-second-order model (Table 1) was obtained. This indicates that chemisorption is the dominant rate-limiting step. This result is reasonable because as indicated previously, the major driving force for the adsorption of AgNPs onto Fe_3_O_4_@PDA is the formation of Ag–catechol bonds through the specific high affinity between AgNPs with PDA on the surface of Fe_3_O_4_@PDA. It is a chelating process and is always controlled either by particle diffusion mechanism or by a second-order chemical reaction^34^.

The adsorption isotherm was obtained after the mixture of Fe_3_O_4_@PDA and AgNPs was shaken for 26 h (Fig. 3c). The correlation coefficient for the Langmuir model is quite high (>0.99) (Table 2), showing a better fit of the Langmuir model with the experimental data as compared to the Freundlich model. The maximum adsorption capacity for AgNPs was 169.5 mg/g, which was higher than those obtained with other adsorbents^10^,^11^,^12^,^13^,^14^,^15^,^17^,^18^ (Table 3). In addition, based on result of *β* value given in Table 2, the mean energy value of GA-AgNPs adsorption determined by the Dubinin-Radushkevich model was 23.57 kJ/mol. Hence, GA-AgNPs are chemisorbed on the Fe_3_O_4_@PDA because a chemical adsorption takes place if a value of adsorption energy is in the range of 8–16 kJ/mol, while a physical adsorption does if it is below 8 kJ/mol^35^.

### Effect of ionic strength

Effect of ion strength on the adsorption of AgNPs was examined by changing the concentration of NaNO_3_ in the 0–50 mM range. Minor effect was observed even when the concentration reached 50 mM (Fig. 3d), indicating that the electrostatic interaction played a minor role in AgNPs adsorption.

### Effect of natural water matrices

The removal ability of Fe_3_O_4_@PDA for GA-AgNPs was investigated with different water matrices (including ultra pure water and river water) spiked with GA-AgNPs. The Jialingjiang River water in Beibei section was collected and used as a practical sample. Before use, the water sample was filtered through a 0.45 μm membrane. The adsorption capacity of Fe_3_O_4_@PDA for GA-AgNPs in river water was almost same as that in ultra pure water, showing the minor effect of river water matrices on removal of GA-AgNPs (Supplementary Table 2). This is probably due to the specific high affinity of AgNPs to PDA through its complexation with catechol group^28^ on Fe_3_O_4_@PDA surface.

### Catalytic reduction of MB by AgNPs-Fe_3_O_4_@PDA

**Figure 4:**
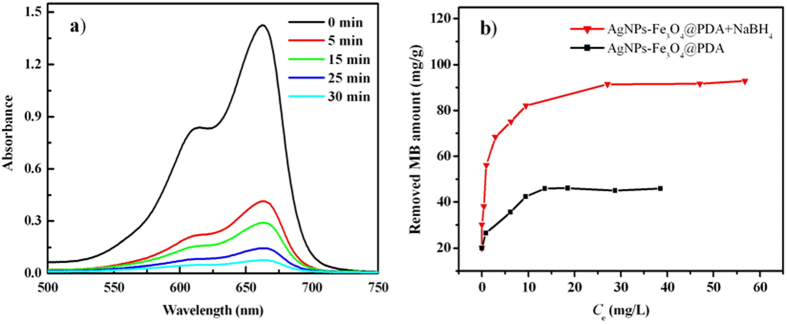
(**a**) Effect of contact time on MB removal by AgNPs-Fe_3_O_4_@PDA/NaBH_4_; (**b**) MB removal by AgNPs-Fe_3_O_4_@PDA/NaBH_4_ and Fe_3_O_4_@PDA/NaBH_4_ systems.

Due to good catalytic activity of AgNPs, the magnetically separated AgNPs-Fe_3_O_4_@PDA was used for the catalytic reduction of MB by NaBH_4_ as reducing agent. MB was selected as a target in present work because of its wide use in coloring paper, temporary hair colorant, dyeing cottons, and so on. Also, it inhibits caspases by oxidation of the catalytic cysteine^36^. The adsorption peak at 663 nm decreased with increase in reaction time in the presence of AgNPs-Fe_3_O_4_@PDA (Fig. 4a). MB was almost totally removed within 30 min, indicating the successful reduction of MB by AgNPs-Fe_3_O_4_@PDA. Also, direct adsorption of MB by Fe_3_O_4_@PDA was observed due to the electrostatic interaction between negatively charged Fe_3_O_4_@ PDA and positively charged MB. The absorption capacity of AgNPs-Fe_3_O_4_@PDA for MB is about 50% of the removed MB amount by AgNPs-Fe_3_O_4_@PDA/NaBH_4_ system (Fig. 4b), indicating that AgNPs-Fe_3_O_4_@PDA exhibits good catalytic performance. pH variance from 4 to 10 has no obvious effect on the catalytic activity of the AgNPs-Fe_3_O_4_@PDA toward MB reduction (Supplementary Figure 4a). Hence, no pH adjustment was needed for MB solution. 5.6 was selected as optimum pH.

The catalytic reduction of MB by AgNPs-Fe_3_O_4_@PDA/NaBH_4_ system can be considered to follow pseudo-first order kinetics because the high initial concentration of NaBH_4_^34^ used in the experiment. Hence, Eq. (1) was used to fit the experimental data:


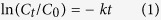


where *C*_0_ and *C*_t_ are the concentration of MB at the initial stage and at time *t*, respectively. *k* represents the reaction rate constant.

The kinetic data obtained with the AgNPs-Fe_3_O_4_@PDA as catalyst (*C*_0_ = 7.5 mg/L, 20 mL of MB solution, 5 mg of catalyst dosage) was fitted to the pseudo-first order kinetics model using a linear fitting and the obtained *k* was 1.44 × 10^−3^/s (Supplementary Figure 4b), which was much higher than those obtained with many other catalysts (Table 4), including AgNPs/P(NIPAM-co-DMA) microgels^37^, Ag nanowire^38^, Sacha inchi (SI) oil templated AgNPs^39^, GO/AgNPs^40^, Ag colloid^41^, Pd/polypyrrole-cellulose^42^, biogenic AuNPs^43^, Sterculia acuminata fruit extract templated AuNPs^44^, Au-PBCGO55^45^, dendrimer encapsulated AgNPs (AgDENs)^46^, dendrimer encapsulated AuNPs (AuDENs)^46^, and Fe_3_O_4_@Tween20@Ag^47^. High catalytic activity of the AgNPs-Fe_3_O_4_@PDA for MB removal is probably attributed to the presence of monodisperse AgNPs on the surface of Fe_3_O_4_@PDA (Fig. 1c), leading to a bigger active contact surface. In addition, the electrostatic interactions between PDA and MB are also in favor of this effect. MB was more easily absorbed on the surface of AgNPs-Fe_3_O_4_@PDA through π–π interaction and hydrogen bonding because of the presence of large amount of functional groups (amino and catechol groups) on PDA layer. Finally, in order to demonstrate whether the AgNPs-Fe_3_O_4_@PDA catalyst obtained from real water matrices could be effective for MB degradation, 5 mg of such AgNPs-Fe_3_O_4_@PDA was added to 20 mL of 7.5 mg/L MB solution in the presence of 0.5 mL of 0.1 M NaBH_4_. The obtained *k* values for two Jianlingjiang river water samples are 1.35 × 10^−3^/s, and 1.38 × 10^−3^/s (Supplementary Figure 4c and 4d), respectively. These results suggest the minor effect of river water matrices on catalytic performance of the obtained AgNPs-Fe_3_O_4_@PDA for the degradation of MB.

### Recyclability and stability of AgNPs-Fe_3_O_4_@PDA

The cycling tests were carried out to study the reusability of AgNPs-Fe_3_O_4_@PDA catalyst. After catalytic reaction, AgNPs-Fe_3_O_4_@PDA was regenerated by treatment using 0.1 M HNO_3_, ethanol and ultra-pure water, then reused in the next catalytic reduction of MB for eight times under the same conditions. Above 85% MB elimination was retained after eight cycles (Supplementary Figure 5), indicating no apparent loss in catalytic activity of the AgNPs-Fe_3_O_4_@PDA for MB removal. Thus, the AgNPs-Fe_3_O_4_@PDA promises good recyclability and great potential in practical applications. Also, the stability of the AgNPs-Fe_3_O_4_@PDA was examined. First, the concentration of the leached iron in the degradation of MB was measured under optimized catalytic conditions and it was 0.22 mg/L. This confirms that the AgNPs-Fe_3_O_4_@PDA catalyst is very stable for MB degradation reaction. Second, we soaked 10 mg of the AgNPs-Fe_3_O_4_@PDA in 1 M HNO_3_ for different time ranging from 6 h to 24 h. Then nitric acid treated adsorbents were used for catalytic reduction of MB. The AgNPs-Fe_3_O_4_@PDA still keep over 95% catalytic activity even treatment in 1 M HNO_3_ environment for 24 h (Supplementary Figure 6). The XRD pattern (Supplementary Figure 7a) shows that AgNPs are still on the surface of Fe_3_O_4_@PDA particles after acid treatment. Further, the sphere-like structure of AgNPs-Fe_3_O_4_@PDA is retained (Supplementary Figure 7b). Such a good stability may be attributed to the presence of PDA layer, which is helpful to protect the Fe_3_O_4_ cores and bind the AgNPs due to high affinity between PDA and AgNPs. These results suggest that the AgNPs-Fe_3_O_4_@PDA is acid resistant and stable in the experimental conditions used.

## Conclusion

In conclusion, we demonstrate that the Fe_3_O_4_@PDA is a promising adsorbent for the extraction and recovery of AgNPs with a maximum adsorptive capacity of 169.5 mg/g. The magnetically separated AgNPs-Fe_3_O_4_@PDA holds highly catalytic activity, good stability and cyclic performance for MB reduction by NaBH_4_. It is potentially useful for the water treatment applications. Notably, present study provides a new way to recover noble metallic NPs for the catalytic removal of contaminants in water.

## Experimental Section

### Chemicals

GA powder, PVA, PVP, HA, citrate, silver nitrate (AgNO_3_), ammonia (NH_3_·H_2_O), sodium borohydride (NaBH_4_), dopamine hydrochloride, six hydrated ferric chloride (FeCl_3_·6H_2_O), and seven ferrous sulfate hydrate (FeSO_4_·7H_2_O) were obtained from Chongqing Taixin Chemical Reagents Company (Chongqing, China). PEI with a molecular weight of 25 000 was purchased from Sigma–Aldrich (Shanghai, China). All chemicals were of analytical grade. The ultra-pure water was used for preparing all solutions.

### Synthesis of GA capped silver nanoparticles

GA stabilized AgNPs were synthesized by reducing AgNO_3_ in water with NaBH_4_^31^. Briefly, 0.2430 g GA powder was added to a flask containing 800 mL of ultra-pure water with vigorous stirring for about 10 min, and a proper amount of 0.02 M AgNO_3_ was added and with constant stirring. Finally, 0.1360 g NaBH_4_ was quickly added with vigorously stirring for 12 h to obtain a yellow GA-AgNPs suspension. The obtained GA-AgNPs was stored at 4 °C for further use, and no aggregation was found for the GA-AgNPs during storage. The AgNPs and AuNPs with other different capping agents were prepared based on the modified procedures previously reported in the literature, which were given in Supporting Information.

### Synthesis of Fe_3_O_4_@PDA core-shell microspheres

The Fe_3_O_4_ microspheres were prepared with a modified coprecipitation method. FeCl_3_·6H_2_O (6.1 g) and FeSO_4_·7H_2_O (4.2 g) were dissolved in 100 mL H_2_O. After being heated to 90 °C with stirring, 25% NH_3_·H_2_O was quickly added with stirring for 30 min. Finally, the black precipitation were washed with water and then dried in vacuum at 60 °C. To coat Fe_3_O_4_ cores with the polydopamine shell, 80 mg of Fe_3_O_4_ and 240 mg of dopamine hydrochloride were dissolved in 50 mL of PBS solution (pH 8.0). After shaking for 24 h at room temperature, the products were separated by using an external magnet, washed with ultra-pure water and ethanol several times, and then dried in an oven at 60 °C for overnight.

### Instrumentation

A XS-105 Mettler Toledo analytical balance (Mettler-Toledo, Switzerland) was used to accurately weigh the amount of the adsorbents. UV-vis spectra were measured with a type UV-2450 spectrophotometer (Shimadzu, Suzhou, China). Fourier transform infrared (FT-IR) spectra were recorded on the Nicolet 170SX instrument (Madison, WI) in the transmission mode using KBr pellets of the sample. Thermogravimetric (TG) data were obtained by the TA-SDTQ 600 (Texas Instruments, Inc., New Castle, DE) in the temperature range from 25 °C to 800 °C at a heating rate of 10 °C/min. The X-ray diffraction (XRD) patterns were recorded using an XD-3 X-ray diffractometer (PuXi, Beijing, China) under the conditions of nickel-filtered Cu K*α* radiation (*λ* = 0.15406 nm) at a current of 20 mA and a voltage of 36 kV. The magnetic property was determined by vibrating sample magnetometry (VSM, HH-15). The zeta potential was measured using a Malvern Instruments Zetasizer Nano-ZS90 (Malvern, UK) instrument. TEM images were obtained on a Tecnai G2 20 (FEI, USA).

### Adsorption experiments and data treatment

The adsorption experiments were carried out in 100 mL stoppered conical flasks. 5 mg portion of Fe_3_O_4_@PDA was added to flask containing 50 mL of GA-AgNPs solution. Subsequently, flasks were shaken at 180 rpm in a thermostatic shaker at 25 °C. After adsorption, the solid adsorbents were separated from the solution with an external magnet. The concentration of the remaining AgNPs suspension was determined using UV–vis spectrophotometer by measuring the changes in the absorbance after adsorption.

For the adsorption kinetic studies, the pseudo-first-order^48^ (equation 2) and pseudo-second-order^49^ (equation 3) models were used to fit the experimental data.


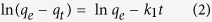



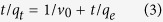


Where *q*_e_ is the equilibrium adsorption capacity (mg/g), *q*_t_ is the adsorption capacity at time *t, k*_1_ (1/min) is the pseudo-first-order adsorption rate constant, and *v*_0_ is the initial adsorption rate [(mg/(g·min)].

Two adsorption isotherms, namely, Langmuir model^13^ (equation 4), Freundlich model^14^ (equation 5), and Dubinin-Radushkevich model^50^ (equation 6) were used to analyze the obtained adsorption data.


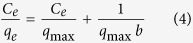



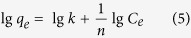



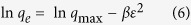


where *C*_e_ is the equilibrium concentration of the studied target solute (mg/L), *q*_max_ (mg/g) is the maximum adsorption capacity, *b* (L/mg) is constant related to energy of adsorption. *k* and *n* are the constants of Freundlich adsorption. *β* is the constant related to adsorption energy (mol^2^/kJ^2^); *ε* is the Polanyi potential that is equal to *RT* ln (1 + 1/*C*_e_). *R* is the gas constant and *T* is the absolute temperature (*K*). Dubinin-Radushkevich isotherm is used to distinguish the physical and chemical adsorption of target in terms of its mean free energy *E* (kJ/mol), which can be calculated by *E* = 1/(2*β)*^0.5^.

### Catalytic reduction experiment

The catalytic reduction experiment was carried out by adding 5 mg of the AgNPs-Fe_3_O_4_@PDA into 20 mL of 7.5 mg/L MB aqueous solution, followed by addition of 0.5 mL of fresh NaBH_4_ aqueous solution (0.1 M). After the mixture was shaken at 180 rpm in a thermostatic shaker at 25 °C for 30 min, the solid AgNPs-Fe_3_O_4_@PDA catalysts were separated from the solution with an external magnet. The concentration of remaining MB solution was determined by measuring the absorbance of the solution at 663 nm. The recovered AgNPs-Fe_3_O_4_@PDA catalysts were washed with 0.1 M HNO_3_ solution, ethanol and ultra-pure water several times, and then used for the next cycle process.

## Additional Information

**How to cite this article**: Wu, M. *et al*. Removal of silver nanoparticles by mussel-inspired Fe_3_O_4_@ polydopamine core-shell microspheres and its use as efficient catalyst for methylene blue reduction. *Sci. Rep.* **7**, 42773; doi: 10.1038/srep42773 (2017).

**Publisher's note:** Springer Nature remains neutral with regard to jurisdictional claims in published maps and institutional affiliations.

## Supplementary Material

Supporting Information

## Figures and Tables

**Table 1:** Kinetic parameters of GA-AgNPs adsorption by Fe_3_O_4_@PDA.

*q*_*e, exp*_ (mg/g)	Pseudo-first-order model			Pseudo-second-order model		
	*q*_*e,cal*_ (mg/g)	*k*_*1*_(1/min)	*r*^2^	*q*_*e,cal*_ (mg/g)	*v*_*0*_[mg/(g·min)]	*r*^2^
83.53	112.95	0.00198	0.6613	96.15	0.1231	0.9330

**Table 2:** Isotherm constants for adsorption of GA-AgNPs onto Fe_3_O_4_@PDA.

*q*_*e, exp*_	Langmuir model			Freundlich model			Dubinin-Radushkevich model		
	*b* (L/mg)	*q*_max_ (mg/g)	*r*^2^	*n*	*k*_*f*_	*r*^2^	*q*_max_ (mg/g)	*β* (mol^2^/kJ^2^)	*r*^2^
160.9	0.7300	169.5	0.9928	4.052	78.12	0.8294	180.33	0.0009	0.9752

**Table 3:** Comparison of the maximum AgNPs adsorption capacity with different adsorbents.

Adsorbent	Adsorption capacity (mg/g)	Refs
biomimetic metal oxides	5.02–54.84	10
The surface modified electrospun PVA membrane	23.83–55.8	11
cellulose-based nanofibers	13.1	12
PVA/Gluten Nanofibres	31.84	13
PEI functionalized carbon spheres	135	14
Amine modified block copolymers	99–117	15
Plasma treated nanofibre membranes prepared by PVA/natural GK	38.62	17
nanofibre membranes prepared by PVA and deacetylated GK	143.4–168.5	18
Fe_3_O_4_@PDA	169.5	This work

**Table 4:** Comparison of kinetic constant (*k*) of different noble metal catalysts in the degradation of MB reported in previous literatures.

Catalyst	Reaction conditions	MB removal (%)	*k* (1/s)	Refs
AgNPs/P(NIPAM-co-DMA)* microgels	40 μL of 1.4 mg/L microgels; 40 μL of 0.37 mg/mL MB; 4 mL of 1 mg/mL NaBH_4_.	100	8.33 × 10^−4^–9.67 × 10^−4^	37
Ag nanowire	Ag nanowire catalytic liquid marbles: 80 μL; 2 mM MB; 0.2 M NaBH_4_.	~100	8.30 × 10^−4^	38
Sacha inchi (SI) oil templated AgNPs	250 μL colloidal AgNPs; 5 mL of 10 mg/L MB with sunlight.	>65	0.46 × 10^−4^	39
graphene oxide (GO)/AgNPs	0.5 mL GO/AgNPs; 1.5 mL of 1 μM MB; 1.00 mL of 0.01 M NaBH_4_.	~90	6.33 × 10^−4^	40
Ag colloid	0.1 mL of 1 mg/mL Ag colloid; 0.1 mL of 1 mM MB; 0.15 mL of 5 mM NaBH_4_.	~83	4.33 × 10^−4^	41
Pd/polypyrrole-cellulose	Pd/polypyrrole-cellulose: 2 mg; 30 μL of 0.4 mg/mL MB.	~69	2.50 × 10^−4^	42
biogenic AuNPs	Glass beads coated with Au NPs; 0.25 mL plant extract; 3 mL of 0.1 mM MB.	~85	6.88 × 10^−4^	43
Sterculia acuminata fruit extract templated Au NPs	30 μL (~29.54 μg) Au NPs; 10^−4^ M MB; 0.1 M NaBH_4_.	100	7.19 × 10^−4^	44
Au-PBCGO55**	30 μL of 0.1 mg/mL Au-PBCGO55; 2 mL of 5 mg/L MB; 1 mL of 0.1 M NaBH_4_.	~70	8.33 × 10^−4^	45
Ag-PBCGO55	30 μL of 0.1 mg/mL Ag-PBCGO55; 2 mL of 5 mg/L MB; 1 mL of 0.1 M NaBH_4_.	~83	1.50 × 10^−3^	45
dendrimer encapsulated AgNPs (AgDENs)	0.15 mL of 0.25 μM AgDENs; 0.205 mL of 15 μM MB; 0.24 mL of 40 mM H_2_O_2_.	~93	2.87 × 10−^4^	46
dendrimer encapsulated AuNPs (AuDENs)	0.15 mL of 0.23 μM AuDENs; 0.205 mL of 15 μM MB; 0.24 mL of 40 mM H_2_O_2_.	~93	3.15 × 10^−4^	46
Fe_3_O_4_@Tween20@Ag	5 mg Fe_3_O_4_@Tween20@Ag; 100 μL of 10 mM MB; 1 mL of 10 mM NaBH_4_.	~70	1.10 × 10^−3^	47
AgNPs-Fe_3_O_4_@PDA	5 mg AgNPs-Fe_3_O_4_@PDA; 20 mL of 7.5 mg/L MB; 0.5 mL of 0.1 M NaBH_4_.	~100	1.44 × 10^−3^	This work

*P(NIPAM-co-DMA): poly (N-isopropylacrylamide-co-2-(dimethylamino)ethylmethacrylate). **50 wt% of pyrene- functionalized poly(methyl methacrylate)-b-poly(dimethylaminoethyl methacrylate) ionic block copolymer-wrapped carbon nanotubes (PBCNTs) with graphene oxide (GO) decorated of AuNPs.
