# Supplementary material for: Removal of silver nanoparticles by mussel-inspired Fe3O4@ polydopamine core-shell microspheres and its use as efficient catalyst for methylene blue reduction
Source: Sci Rep. 2017 Feb 16;7:42773. doi: 10.1038/srep42773 (PMC5311861; doi:10.1038/srep42773)
Supplement: Supporting Information [file srep42773-s1.pdf]

Supporting Information

Removal of silver nanoparticles by mussel-inspired Fe_3O_4@ polydopamine core-shell microspheres and its use as efficient catalyst for methylene blue reduction

Maoling Wu, Yinying Li, Rui Yue, Xiaodan Zhang, Yuming Huang*

The Key Laboratory of Eco-environments in Three Gorges Reservoir Region, Ministry of Education, College of Chemistry and Chemical Engineering, Southwest University, Chongqing 400715, China.

Synthesis of AgNPs capped with PVA, PVP, HA, Cit and PEI

PVP capped AgNPs were prepared according to the previously reported method[1] with a slight modification. Briefly, 4 mL of 0.02 M $AgNO_3$ were added to 400 mL of 0.25% PVP (wt), followed by adding 0.1 g $NaBH_4$. The resulting mixture was stirred vigorously for 12 h.

PVA capped AgNPs were synthesized by reducing $AgNO_3$ in water with $NaBH_4$. Briefly, 4 mL of 0.02 M $AgNO_3$ were added to 400 mL of 1.25% PVA (wt), followed by adding 0.1 g $NaBH_4$ with vigorously magnetic stirring for 12 h.

HA capped AgNPs were developed according to the previously reported method[2] with a slight modification. Briefly, 4 mL of 0.02 M $AgNO_3$ were added to 400 mL of 0.0012% HA (wt), followed by adding 32 mg $NaBH_4$ with vigorously magnetic stirring for 12 h.

Cit capped AgNPs were prepared according to the previously reported method[3] with a slight modification. Briefly, 4 mL of 0.02 M $AgNO_3$ were added to 400 mL of 0.04% trisodium citrate (wt), followed by adding 0.64 g $NaBH_4$ with vigorously magnetic stirring for 12 h.

PEI capped AgNPs were synthesized according to the previously reported method[4] with a slight modification. Briefly, 10 mL of 0.02 M $AgNO_3$ were added to 200 mL of 0.01% PEI (wt), followed by adding 18 mg $NaBH_4$ with vigorously magnetic stirring for 12 h.

Synthesis of AuNPs capped with Cit, PVP, and GA

Cit capped AuNPs were prepared based on the previously reported method[3] with a slight modification. Briefly, 5 mL of 10 mg/mL tetrachloroauric (III) acid hydrate ($AuCl_3{\cdot}HCl{\cdot}4H_2O$) were added to 500 mL of 0.02% trisodium citrate

solution, followed by dropwise adding 5 mL of 10 mg/mL $NaBH_4$ solution under magnetic stirring for 8 h.

PVP capped AuNPs were synthesized according to the previously reported method[5] with a slight modification. In brief, 5 mL of 0.045 M trisodium citrate solution were added to 500 mL of 0.1% PVP, and then 5 mL of 10 mg/mL tetrachloroauric (III) acid hydrate were added. The resultant mixture was heated at 75 °C for 10 min in a water bath.

GA capped AuNPs were prepared according to the previous method[6] with a slight modification. Brifely, 5 mL of 10 mg/mL tetrachloroauric (III) acid hydrate were added to 500 mL of 0.04% GA, followed by dropwise adding 5 mL of 10 mg/mL $NaBH_4$ under magnetic stirring for 8 h.

Supporting References

1. El Badawy, A. M., Hassan, A. A., Scheckel, K. G., Suidan, M. T., Tolaymat, T. M. Key factors controlling the transport of silver nanoparticles in porous media. *Environ. Sci. Technol.* **47**, 4039−4045 (2013).
2. Croteau, M.-N., Misra, S. K., Luoma, S. N., Valsami-Jones, E. Silver bioaccumulation dynamics in a freshwater invertebrate after aqueous and dietary exposures to nanosized and ionic Ag. *Environ. Sci. Technol.* **45**, 6600–6607 (2011).
3. Kumar, J., Mallampati, R., Adin, A., Valiyaveettil, S. Functionalized carbon spheres for extraction of nanoparticles and catalyst support in water. *ACS Sustainable Chem. Eng.* **2**, 2675−682 (2014).
4. Lee, H. J. *et al.* Antimicrobial polyethyleneimine-silver nanoparticles in a stable colloidal dispersion. *Colloid Surface B* **88**, 505−511 (2011).
5. Zheng, H., Huang, B., Hu, J., Gong, Y. Synthesis and characterization of gold nanoparticles with various diameters. *Acta Scientiarum Naturalium Universitatis Pekinensis* **47**, 777−782 (2011).
6. de Barros, H. R. *et al.* Stability of gum arabic-gold nanoparticles in physiological simulated pHs and their selective effect on cell lines. RSC Adv. **6**, 9411−9420 (2016).

Table S1. Adsorption capacities of AgNPs with different capping agents by Fe_3O_4@PDA

AgNPs with different capping agents	Zeta potential at pH 10 (mV)	Hydrodynamic diameter (nm)	Adsorption capacities (mg/g)
GA-AgNPs	-29.2	72.9	77.68
PVA-AgNPs	-11.1	66.5	33.54
PVP-AgNPs	-19.4	44.0	29.10
HA-AgNPs	-35.6	26.2	21.03
Cit-AgNPs	-33.4	39.4	10.82
PEI-AgNPs	28.4	84.5	23.98

Table S2. Adsorption capacities of GA-AgNPs by Fe_3O_4@PDA in different water matrices

Matrices	Adsorption capacities (mg/g±SD, *n*=2)
ultra pure water	77.68±2.73
Jialingjiang river water 1#	77.58±1.25
Jialingjiang river water 2#	78.36±1.73

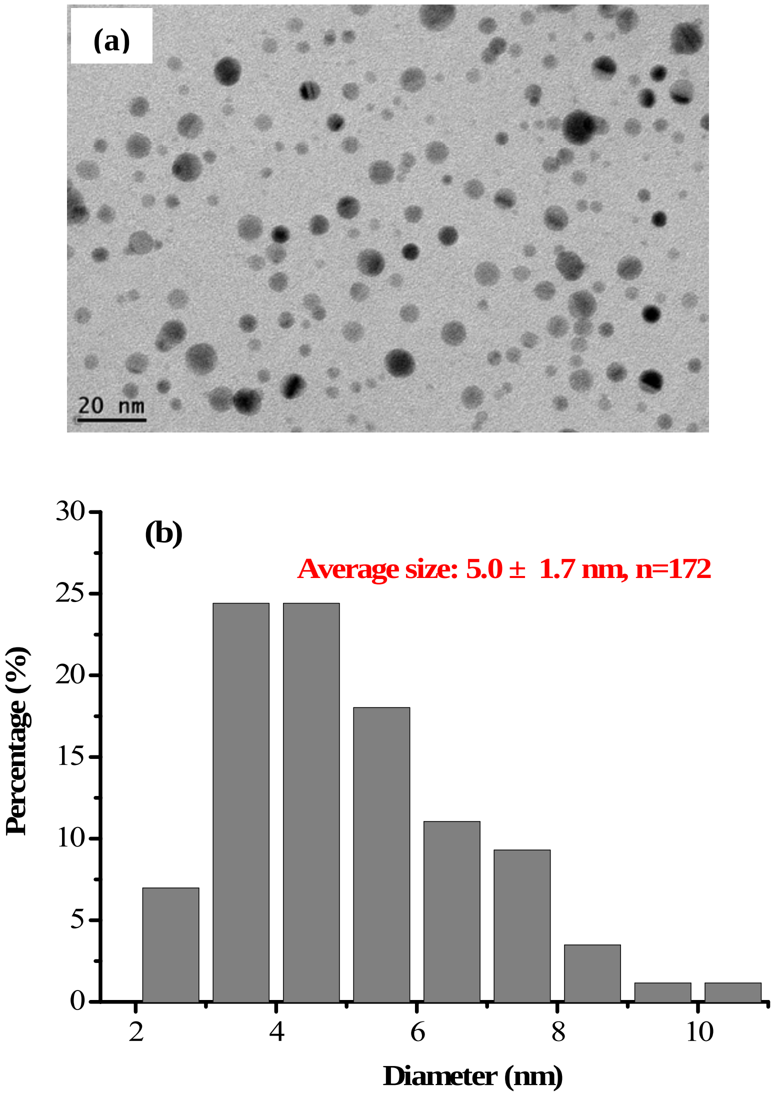

Figure S1. (a) TEM image of GA-AgNPs. (b) The size distribution histogram of GA-AgNPs.

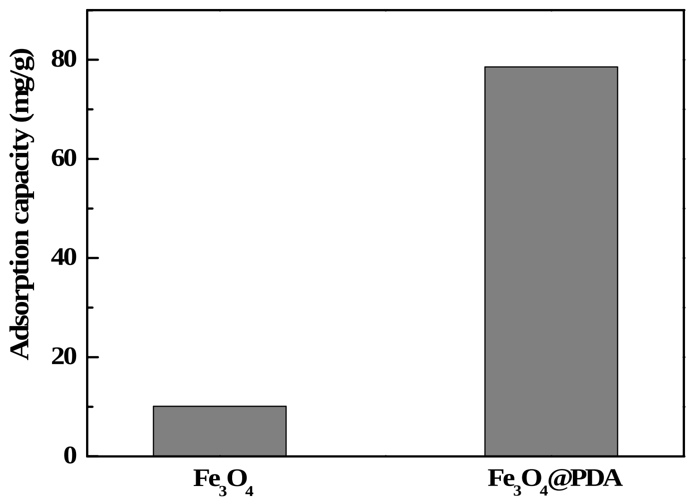

Figure S2. Adsorption comparison of Fe_3O_4 and Fe_3O_4@PDA for GA-AgNPs. Reaction conditions: 5 mg of adsorbents, 50 mL of 10.8 mg/L GA-AgNPs solution, pH 10.0, adsorption time 26 h.

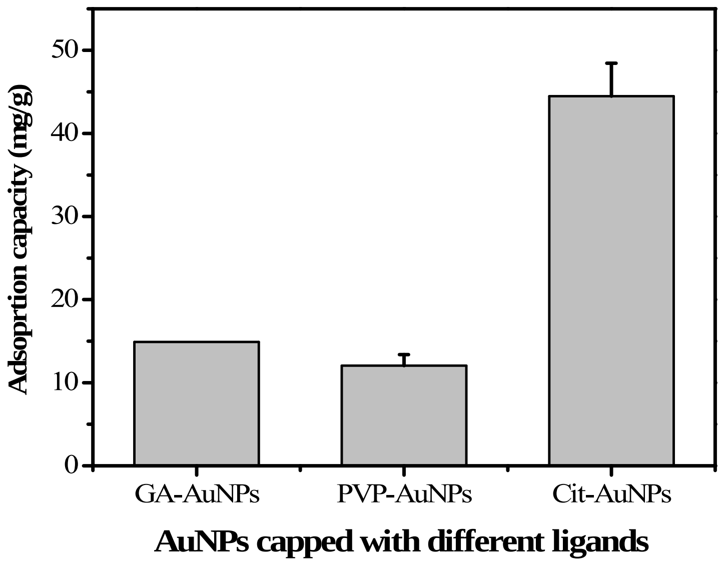

Figure S3. Adsorption capacities of Fe_3O_4@PDA for AuNPs capped with different ligands. Reaction conditions: 5 mg of adsorbents, 50 mL of 56.82 mg/L AuNPs solution, adsorption time 26 h. pH values were 7.0, 7.0, and 9.6 for GA-AuNPs, PVP-AuNPs, and Cit-AuNPs, respectively. Error bars represent one standard deviation for two measurements.

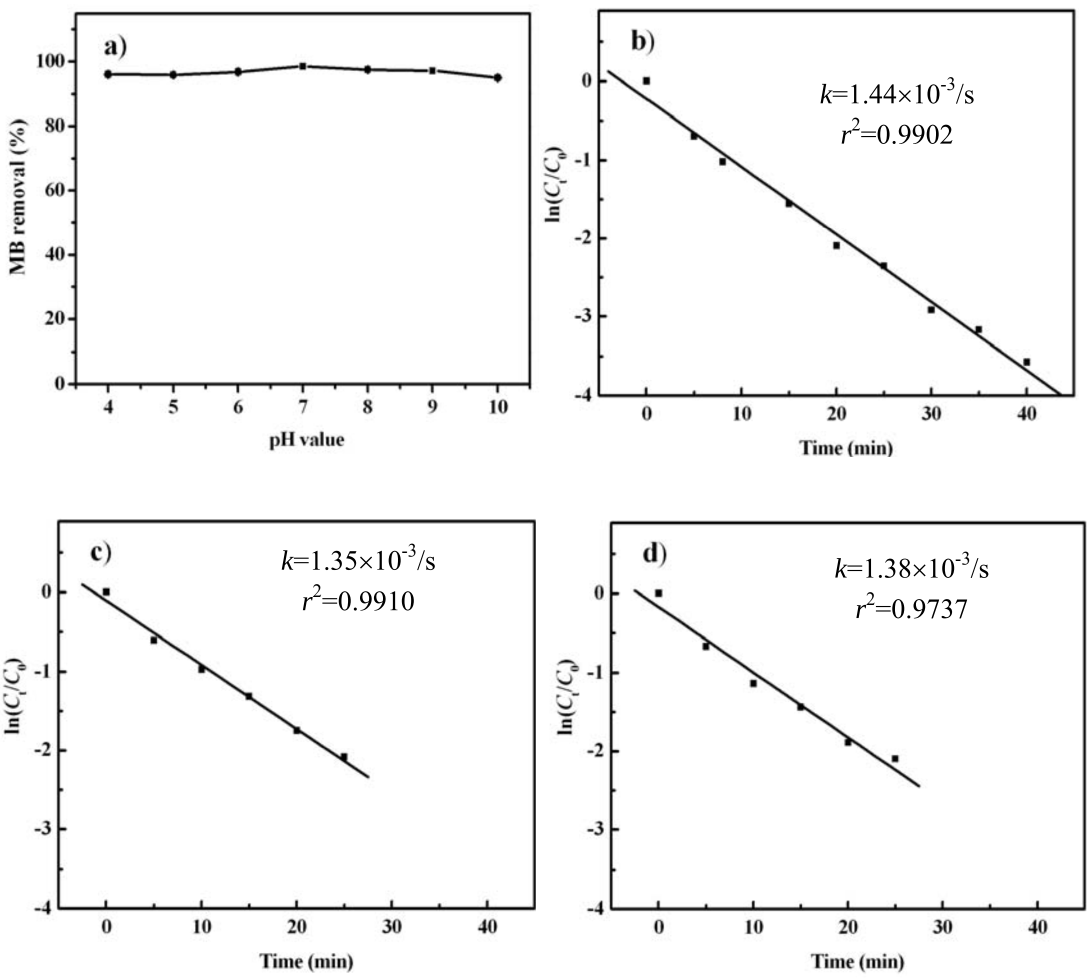

Figure S4. (a) Catalytic reduction of MB by AgNPs-Fe_3O_4@PDA/$NaBH_4$ system at different pH from 4 to 10. Experimental conditions: 20 mL of 7.5 mg/L MB, 5 mg AgNPs-Fe_3O_4@PDA, 0.5 mL of fresh $NaBH_4$ aqueous solution (0.1 M), 30 min of reaction time. Plot of ln(C_t/C_0) against reaction time for the catalytic reduction of MB in the presence of AgNPs-Fe_3O_4@PDA in (b) ultra pure water, (c) Jialingjiang river water sample 1#, and (d) Jialingjiang river water sample 2#. Experimental conditions: 20 mL of 7.5 mg/L MB, 5 mg AgNPs-Fe_3O_4@PDA, 0.5 mL of fresh $NaBH_4$ aqueous solution (0.1 M), pH 5.6.

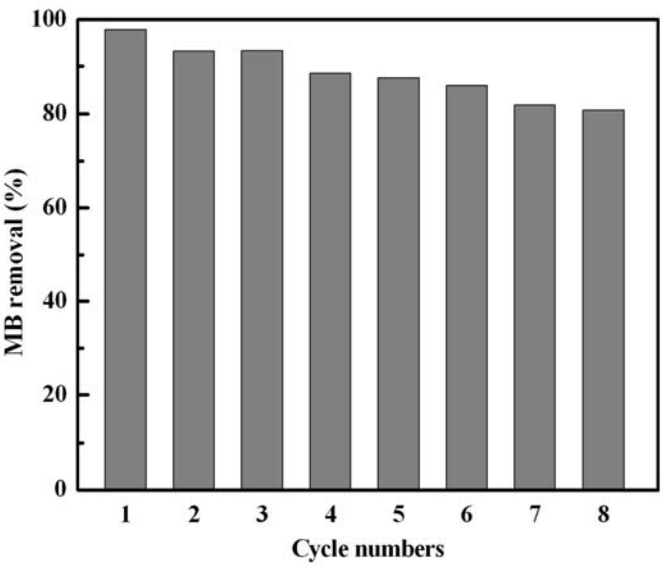

Figure S5. The recyclability of AgNPs-Fe_3O_4@PDA for the catalytic removal of MB. Reaction conditions: 20 mL of 7.5 mg/L MB, 5 mg AgNPs-Fe_3O_4@PDA, 0.5 mL of fresh $NaBH_4$ aqueous solution (0.1 M), pH 5.6, 30 min of reaction time.

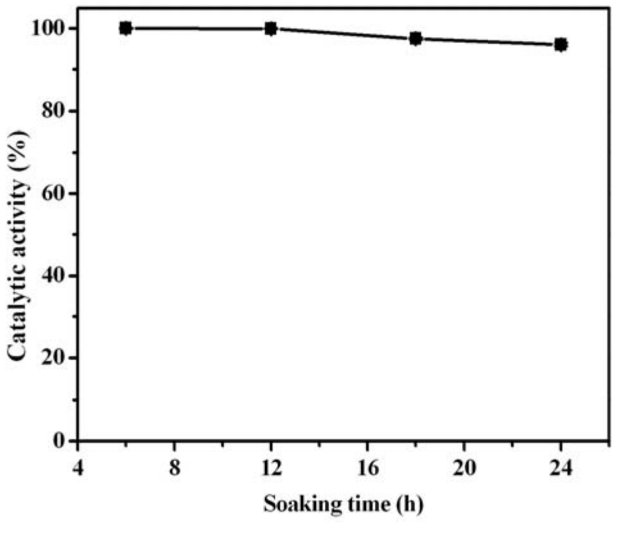

Figure S6. Effect of soaking time on catalytic activity of AgNPs-Fe_3O_4@PDA for MB reduction. Catalytic reaction conditions: 20 mL of 7.5 mg/L MB, 5 mg AgNPs-Fe_3O_4@PDA, 0.5 mL of fresh $NaBH_4$ aqueous solution (0.1 M), pH 5.6, 30 min of reaction time.

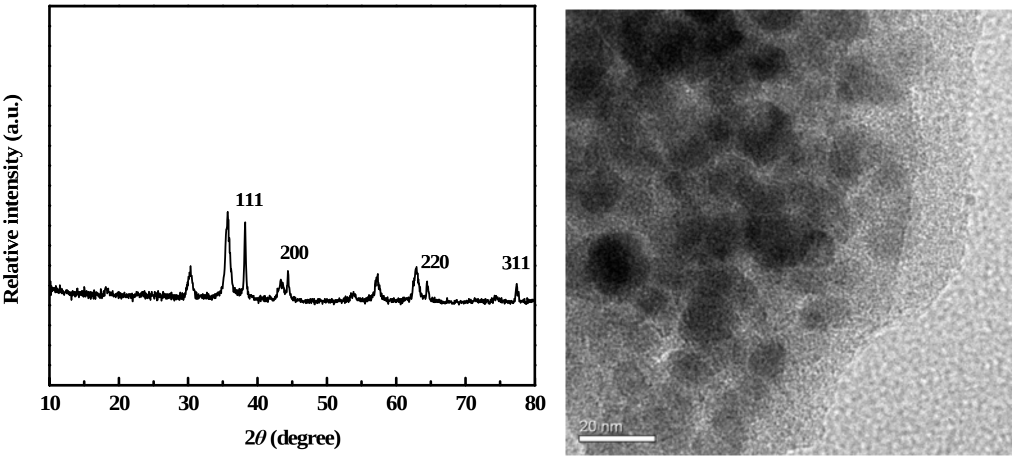

(a) **(b)**

Figure S7. XRD pattern and TEM image of AgNPs-Fe_3O_4@PDA (scale bar=20 nm) treated in 1 M HNO_3 for 24 h.
